# The Role of Leucoyte-Derived Free Oxygen Radicals in the Pathogenesis of Experimental Acute Pancreatitis

**DOI:** 10.1155/1995/36343

**Published:** 1995

**Authors:** E. Akpinar, I. Özden, N. Savci, A. Emre

**Affiliations:** Hepatopancreatobiliary Surgery Unit Department of General Surgery Istanbul Faculty of Medicine Istanbul Turkey

## Abstract

The role of free oxygen radicals in experimental acute pancreatitis induced by common bile duct ligation was investigated by measuring malondialdehyde levels in the rat pancreas. Also, the potential role of leucocytes as the source of free oxygen radicals was tested by inducing leukopenia with methotrexate. The malondialdehyde levels in the control, pancreatitis and pancreatitis + methotrexate groups were 9.6 ± 2.0, 44.8 ± 11.4, and 25.6 ± 5.0 nmol malondialdehyde/ g pancreas tissue respectively. The corresponding histopathological severity scores were 0.5 ± 0.7, 8.1 ± 1.2 and 3.7 ± 1.1. The results suggest that the leucocyte may be an important source of free oxygen radicals in this experimental model.


*HPB Surgery*, 1995, Vol. 8, pp. 237–239
Reprints available directly from the publisher
Photocopying permitted by license only

© 1995 OPA (Overseas Publishers Association)
Amsterdam B.V. Published in The Netherlands
by Harwood Academic Publishers GmbH
Printed in Malaysia

### The Role of Leucoyte-Derived Free Oxygen Radicals in the Pathogenesis of Experimental Acute Pancreatitis

E. AKPINAR, I. ÖZDEN, N. SAVCI and A. EMRE

Hepatopancreatobiliary Surgery Unit, Department of General Surgery
Istanbul Faculty of Medicine, Istanbul, Turkey

*(Received 10 January 1994)*

The role of free oxygen radicals in experimental acute pancreatitis induced by common bile duct ligation was investigated by measuring malondialdehyde levels in the rat pancreas. Also, the potential role of leucocytes as the source of free oxygen radicals was tested by inducing leukopenia with methotrexate. The malondialdehyde levels in the control, pancreatitis and pancreatitis + methotrexate groups were $9.6 \pm 2.0$, $44.8 \pm 11.4$, and $25.6 \pm 5.0$ nmol malondialdehyde/g pancreas tissue respectively. The corresponding histopathological severity scores were $0.5 \pm 0.7$, $8.1 \pm 1.2$ and $3.7 \pm 1.1$. The results suggest that the leucocyte may be an important source of free oxygen radicals in this experimental model.

KEY WORDS: Acute pancreatitis oxygen radicals leucocyte

### INTRODUCTION

Free oxygen radicals (FORs) are metabolic by-products which are rapidly inactivated by scavenger systems. Under pathological conditions, the production rate surpasses the inactivation capacity of the cell and these radicals react with cellular macromolecules, most importantly, membrane lipids and cause cell injury.

Sanfey *et al.* have shown that FORs play a role especially in early pancreatitis and have hypothesized that, FORs, which are increased in all pancreatitis models, originate from pancreatic chymotrypsinogen activity and xanthine dehydrogenase (XD)-derived xanthine oxidase (XO)[1]. Gough *et al.* have also shown that FORs are increased in acute pancreatitis and suggested that the leucocyte is the major source of FORs[2]. Wiesner *et al.*[3] and Rutledge *et al.*[4] in two different models of acute pancreatitis, confirmed the role of the FORs but observed no amelioration with allopurinol, a xanthine oxidase inhibitor. Consequently, these groups argued that FORs originate, not from the xanthine oxidase but from leucocytes and endothelial cells. Accordingly, Gilrane *et al.* have reported that antineutrophil serum has beneficial effects in cerulein-induced pancreatitis[5]. On the other hand, Sarr *et al.* have shown that leucocytes are not essential in pancreatitis in an in vitro perfusion model[6]. The topic is still controversial and further data are needed. In this study we investigated the role of leucocytes in acute pancreatitis induced by common bile duct ligation.

### MATERIALS AND METHODS

This study was conducted at the Center for Experimental Medical Research & Application (DETAM) of Istanbul University. Adult, female Wistar albino rats (180–220 g) were used. The animals were provided with standard laboratory chow and water ad libitum.

Three groups were established (n = 10):
1) Control group
2) Acute pancreatitis group
3) Acute pancreatitis group treated with methotrexate

Address for correspondance: Prof. Ali Emre Ataköy 9-10 Mah., A7-9-5 34750, ISTANBUL/TURKEY.

Blood samples were obtained from all animals for basal leucocyte counts. Group 1 underwent laparatomy only. Acute pancreatitis was induced by ligating the common bile duct at its point of entry into the duodenum. Group III received methotrexate at a daily dose of 2.5 mg/kg intraperitoneally starting 12 hours before bile duct ligation.

Twenty-four hours after laparotomy, the animals were sacrificed. Blood samples were taken by cardiac puncture for leucocyte counts and amylase measurements. Each pancreas was divided for histopathological examination and malondialdehyde (MDA) measurement (stored at − 70°C until the assay).

Amylase was measured by the amyloclastic method of Caraway[7]. Briefly, 2.5 mL of disodium phosphate/benzoic acid-buffered (PH 7.0) starch substrate (0.4 g/l) was heated in a 37°C water bath for 15 minutes and 50 µL of serum was added. After 7.5 minutes, the tube was removed from the bath and 1.5 mL of water and 2.5 mL of iodine solution (0.01 M/L) were added. Then the solution was diluted to 25 mL and absorbance against water was read at 660 mm. Since iodine reacts with the remaining starch, the amount of amylase in the serum is inversely proportional to the intensity of the color generated.

Malondialdehyde levels (MDA) were measured by the thiobarbituric acid method[8]. Briefly, tissue samples were homogenized in 9 volumes of ice-cold 5% trichloroacetic acid solution (TCA) using an Ultraturrax homogenizer. One mililiter of homogenate was added to 1 mL of 0.67% thiobarbituric acid (TBA) solution. The mixture was kept at 100°C for 10 minutes. The absorbances were measured at 532 nm. Malondialdehyde levels were calculated by using the extinction coefficient $1.56 \times 10^5$.

Leucocyte counts were performed manually. The sections were graded from 0 to + + + with respect to edema, hemorrhage, necrosis, lipid necrosis and leucocytic infiltration[9]. Consequently a score between 0 and 15 was assigned to each sample. The results were expressed as mean +/− standard error and evaluated by Student's t-test or the Mann-Whitney U-test. A p value less than 0.05 was considered significant.

### RESULTS

The results are summarized in table 1. Amylase and MDA (a parameter reflecting lipid peroxidation by FORs) levels were significantly increased in Groups 2 and 3, but the levels were higher in Group 2. The same pattern was observed in the histopathological grading. In the AP + MTX group, leucocyte infiltration especially was limited in comparison with the AP group.

### DISCUSSION

It is widely accepted that FORs play a significant role in acute pancreatitis. However, the source of these FORs is controversial. In their studies in the ex vivo perfused dog pancreas, Sanfey *et al.*[1,10] and Sarr *et al.*[6] have suggested that these are produced by xanthine oxidase. However, others have obtained contradictory data in other models[2–5] and argued that the leucocyte is the major source of FORs in acute pancreatitis. Further, some authors have used the degree of leucocyte infiltration as a criterion for the evaluation of severity and prognosis and have shown that higher levels of infiltration reflect a worse prognosis[11].

In this study, methotrexate treatment was used to induce leucopenia; the MDA levels and histological severity of pancreatitis were investigated under this condition. Cytostatics have been used previously in experimental acute pancreatitis. Johnson *et al.* have reported beneficial effects of 5-FU. They attributed this to the inhibition of protein synthesis in the pancreas and the induction of a rest state[12]. Korbous *et al.* have tried various cytostatics and obtained parallel re-

**Table 1** Evaluation parameters

| | *Control* | *Pancreatitis (AP)* | *Pancreatitis + Methotrexate (AP + MTX)* |
|---|---|---|---|
| Amylase (u/L) | 75 +/− 12 | 198 +/−33 | 108 +/−15* |
| Leucocyte count (cells/mm³) | 10200 +/− 1700 | 17600 +/−2000 | 1520 +/− 760* |
| MDA (nmol/g) | 9.6 +/− 2.0 | 44.8 +/−11.4 | 25.6 +/− 5.0* |
| Pathological score | 0.5 +/− 0.1 | 8.1 +/− 1.2 | 3.7 +/− 1.1* |

$P < 0.05$ (Group AP versus Group AP + MTX)

sults[13]. In our study, tissue MDA levels were increased in the pancreatitis groups in comparison with the control group. This finding confirms that FORs play a significant role in acute pancreatitis. In the group rendered leucopenic by methotrexate, the pancreatitis was less severe and more importantly, MDA levels were significantly lower in comparison with the untreated group. In other words, leucopenia was accompanied by suppression of increased lipid peroxidation and prevention of severe pancreatitis. Our results suggest that a significant fraction of the FORs which play a role in the pathogenesis of pancreatitis are derived from leucocytes. Induction of leucopenia would not be feasible in the clinical setting. However, agents which modulate the leucocyte-pancreas tissue interaction (for example, antibodies against adhesion molecules) may have exciting applications.

### REFERENCES

1. Sanfey, H., Sarr, M. G., Bulkley, G. B. and Cameron, J. L. (1986) Oxygen-derived free radicals and acute pancreatitis: A review. *Acta Physiol. Scand.*, **548** (suppl), 109–118..
2. Gough, D. B., Boyle, B., Joyce, W. P., Delaney, C. P., McGeeney, K. F., Gorey, T. F. and Fitzpatrick, J. M. (1990) Free radical inhibition and serial chemiluminescence in evolving experimental pancreatitis. *Brit. J. Surg.*, **77**, 1256–1259.
3. Wisner, J., Green, D., Ferrel, L. and Renner, I. (1988) Evidence for a role of oxygen-derived free radicals in the pathogenesis of cerulein-induced acute pancreatitis in rats. *Gut*, **29**, 1516–1523.
4. Rutledge, P. L., Saluja, A.K., Powers, R, E. and Steer, M. L. (1987) Role of oxygen-derived free radicals in diet-induced hemorrhagic pancreatitis in mice. *Gastroenterology*, **93**, 41–47.
5. Gilrane, T. B., Glover, W., Granger, D. N., Holt, S. L. and Powers, R. E. (1989) A temporal role of neutrophils in the pathogenesis of cerulein-induced acute pancreatitis. *Pancreas*, **4**, 617–621.
6. Sarr, M G., Bulkley, G. B. and Cameron, J. L. (1987) The role of leukocytes in the production of oxygen-derived free radicals in acute experimental pancreatitis. *Surgery*, **101**, 292–296.
7. Kaplan, A. and Szabo, L. L. (1983) Enzymes. In Clinical Chemistry: Interpretation and Techniques. Eds. Kaplan *et al.* Second Edition, pp 207–208, Philadelphia, Lea Febiger.
8. Pompella, A., Maellaro, E., Casini, A. F., Ferrali, M., Ciccoli, L. and Comporti, M. (1987) Measurement of lipid peroxidation in vivo: A comparison of different procedures. *Lipids*, **22**, 206–211.
9. Armstrong, C. P., Taylor, T. V. and Torrance, H. B. (1985) Pressure, volume and the pancreas. *Gut*, **26**, 615–624.
10. Sanfey, H., Bulkley, G. and Cameron, J. L. (1984) The role of oxygen-derived free radicals in the pathogenesis of acute pancreatitis. *Ann. Surg.*, **200**, 405–413.
11. Schölmerich, E., Schumichen, C., Lausen, M., Gross, V., Leser, H. G., Lay, L., Farthmann, E. H. and Gerok, W. (1991) Scintigraphic assessment of leukocyte infiltration in acute experimental pancreatitis using technetium 99m-hexamethylpropyleneamine oxine as leukocyte label. *Dig. Dis. Sci.*, **36**, 65–70.
12. Johnson, R. M., Barone, R. M., Newson, B. L., DasGupta, T. K. and Nyhus, L. M. (1973) Treatment of experimental pancreatitis with 5-fluorouracil. *Am. J. Surg.*, **125**, 211–221.
13. Korbova, L., Kohout, J., Malis, F., Balas, V., Cizkova, J., Marek, J. and Cihak, A. (1977) Inhibitory effect of various cytostatics and cycloheximide on acute pancreatitis in rats. *Gut*, **18**, 913 –918.

